# Effective Dose of Radon 222 Bottled Water in Different Age Groups Humans: Bandar Abbas City, Iran

**DOI:** 10.5539/gjhs.v8n2p64

**Published:** 2015-06-05

**Authors:** Yadolah Fakhri, Amir Hossein Mahvi, Ghazaleh Langarizadeh, Yahya Zandsalimi, Leila Rasouli Amirhajeloo, Morteza Kargosha, Mahboobeh Moradi, Bigard Moradi, Maryam Mirzaei

**Affiliations:** 1 Social Determinants in Health Promotion Research Center, Hormozgan University of Medical Sciences, Bandar Abbas, Iran; 2 Department of Environmental Health Engineering, Faculty of Health, Tehran University of Medical Sciences, Tehran, Iran; 3 Food and Drugs Research Center, Bam University of Medical Sciences, Bam, Iran; 4 Environmental Health Research Center, Kurdistan University of Medical Sciences, Sanandaj, Iran; 5 Department of Environmental Health Engineering, School of Public Health, Qom University of Medical Sciences, Qom, Iran; 6 Department of Environmental Health Engineering, School of Public Health, Shahid Beheshti University of Medical sciences, Tehran, Iran; 7 Department of Health Public, Kermanshah University of Medical Sciences, Kermanshah, Iran; 8 Jahrom University of medical sciences, Jahrom, Iran

**Keywords:** Radon 222, effective dose, age groups, bottled water, Bandar Abbas

## Abstract

Radon 222 is a natural radioactive element with a half-life of 3.8 days. It is odorless and colorless as well as water-soluble. Consuming waters which contain high concentration of ^222^Rn would increase the effective dose received by different age groups. It would also be followed by an increased prevalence of cancer. In this research, 72 samples of the most commonly used bottled water in Bandar Abbas were collected in 3 consecutive months, May, June and July of 2013. Concentration ^222^Rn of was measured by radon-meter model RTM166-2. The effective dose received by the 4 age groups, male and female adults as well as children and infants was estimated using the equation proposed by UNSCEAR. The results revealed that the mean and range concentration of ^222^Rn in bottled waters were 641±9 Bq/m^3^ and 0-901 Bq/m^3^, respectively. The mean concentration of ^222^Rn in the well-known Marks followed this Zam Zam>Bishe>Koohrng>Dassani>Christal>Polour>Damavand>Sivan. Infants were observed to receive a higher effective dose than children. The highest and lowest effective dose received was found to belong to male adults and children, respectively.

## 1. Introduction

Radon 222 (^222^Rn) is a natural radioactive element with a half-life of 3.8 days. It is colorless and odorless. By emission alpha particles during its decay, it can cause lung, blood and gastric cancer in the long run (Colmenero Sujo et al., 2004; Nagaraja et al., 2003; Smith, Zhang, & Field, 2007). Due to a high ionization power and in terms of internal radiation, an alpha particle is at the top list of hazards as compared to all other particles (Hamanaka, Shizuma, Wen, Iwatani, & Hasai, 1998). ^222^Rn and its daughters, Po214 and Po218 are among the main and final products of the decay of Uranium 235 (^235^U) chain, which can spread out of different sources such as surface and groundwater water, soil, igneous or sedimentary rocks (Kam & Bozkurt, 2007; Oner, Yalim, Akkurt, & Orbay, 2009). In the USA, ^222^Rn is considered as the second cause of mortalities due to lung cancer, smoking being the first (Thompson, Nelson, Popkin, & Popkin, 2008). ^222^Rn has a high water-solubility. A decrease in water temperature would lead to a rise of its solubility (Kam & Bozkurt, 2007). Due to the more contact of groundwater water with igneous and sedimentary rocks, concentration of radioactive contents in these waters can be higher than surface water sources (Akawwi, 2014; Ali, Khan, Akhter, Khan, & Waheed, 2010; Kam & Bozkurt, 2007; Rangela et al., 2002). Moreover, concentration ^222^Rn in groundwater water sources is two to three times as high as other radioactive contents (Forte, Rusconi, Cazzaniga, & Sgorbati, 2006). People are constantly either externally or internally exposed to radioactive materials especially ^222^Rn through respiration and drinking water (M.Rožmaric, Rogic, Benedik, & M.Štrok, 2012; United Nations Scientific Committee on the Effects of Atomic Radiation, 2000). A body of research has indicated that consuming waters which contain high concentrations of ^222^Rn would raise the received effective dose and also the probability of affliction with lung and gastric cancer (Rožmarić, Rogić, Benedik, & Štrok, 2012). The standard set by World Health Organization (WHO) for the ^222^Rn of drinking water is 100,000 Bq/m^3^ (Risica & Grande, 2000). The same standard limit set by the United States Environmental Protection Agency (EPA) is 11,000 Bq/m^3^ (K. Somlai et al., 2007). WHO and the European Committee have proposed the annually received effective dose of ^222^Rn to be 0.1 mSv/y (K. Somlai et al., 2007). Consumption of bottled water has been on the rise in the last 30 years (Karamanis, Stamoulis, & Ioannides, 2007). Bottled waters are divided in two groups of mineral water and bottled water (Bharath et al., 2003). A great many research has indicated that the radioactive contents of bottled water especially mineral water are much more than that of the public distribution network (J. Somlai et al., 2002). According to world statistics, Iran ranks 14^th^ as for the consumption of mineral water (Kiliari & Pashalidis, 2008). Knowledge about the radioactive content of drinking water especially bottled water is minimal in Iran. In Bandar Abbas due to its hot and humid climate and less trust to the quality of tap water, people vastly rely on bottled drinking water. Therefore, the present research attempted to measure concentration of ^222^Rn in 8 of the most famous Marks of bottled water in Bandar Abbas. Eventually, the effective dose received from drinking water was measured across male and female adults, children and infant age groups. These values were later compared with the standard limits.

## 2. Materials and Methods

### 2.1 Number of Samples and Measurement Concentration of ^222^Rn

Samples were selected in three stages and within three months: May, June and July of 2013. Each month corresponded to one stage of data collection. In each stage, samples of the 8 most famous and widely-used bottled water Marks in Bandar Abbas city were randomly collected from stores: Dassani, Bishe, Polour, Zam-Zam, Damavand, Chrystal, Sivan and Koohrang. From each Marks, three 1.5-liter bottles were collected and kept at the temperature of 4 to 6°C in a chemistry lab of Bandar Abbas Water and wastewater Company (Binesh, Mohammadi, Mowlavi, & Parvaresh, 2010). On the whole in the three stages, 72 bottled water samples were selected as from among the most widely-used Marks of the city.

**Figure 1:**
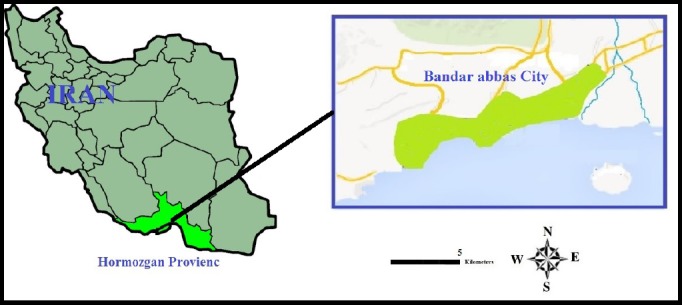
Location of Bandar Abbas city in Hormozgan province and Iran

**Figure 2:**
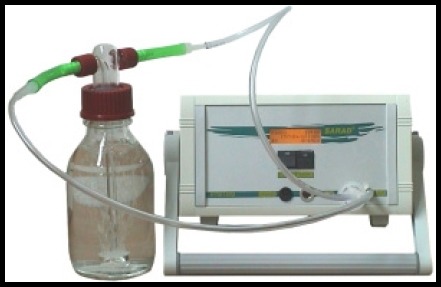
Measurement concentration ^222^Rn by of Radon-meter model RTM1688-2

Considering the effect of temperature on the radiation of ^222^Rn, before the measurement the temperature of all samples was made uniform (12 °C) (Ishikawa, Tokonami, Yoshinaga, & Narazaki, 2005; K. Somlai et al., 2007). Concentration of ^222^Rn was measured by Radon meter model RTM166-2 made by the sarad company (Figure 2). Sensitivity of this instrument in 150 minutes of constant measurement was 6.5 cts (min×KBq/m^3^) (Ursulean, Corețchi, Chiruță, & Virlan, 2012). High sensitivity along with a spectral analysis of alpha can cut down on the response time even in low concentration. The measurement concentration of ^222^Rn in the samples was done following the instructions provided by Sarad Company. Similarly, the 2 hour mean concentration of ^222^Rn was recorded and analyzed for all the samples (GmbH, June 2007).

### 2.2 Estimating the Annually Effective Dose

Once an individual consumes a water containing ^222^Rn, the alpha spread out during its decay can damage the DNA of the inner-gastric cells. On the other hand, by penetrating the gastric membrane, it can enter blood and can spread all throughout the body (Organization, 2004). Therefore, in order to estimate the annually received effective dose of ^222^Rn through drinking bottled water, an equation is proposed by the United Nations Scientific Committee on the Effects of Atomic Radiation (UNSCEAR) (J. Somlai et al., 2002):


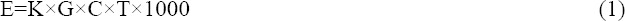


In this equation: E is the annually received effective dose (in mSv/y), K; the conversion coefficient concentration of ^222^Rn to effective dose (Sv/Bq), G; the daily consumed water (l/d), C; the concentration of ^222^Rn (Bq/l); T; the time span of water consumption (365 days) and 1000; the conversion coefficient of Sv to mSv.

K coefficient of male and female adults (17-65 years of age), children (4-14 years of age) and infants (younger than 2 years of age) is: 18×10^-9^ Sv/Bq, 26×10^-9^ Sv/Bq and 35×10^-9^ Sv/Bq, respectively (Binesh et al., 2010; United Nations. Scientific Committee on the Effects of Atomic Radiation, 2009; K. Somlai et al., 2007). There is a limited world information about the daily amount of water consumed. A myriad of research has indicated that the amount of people’s consumed water is less than 2 liters a day. It varies across different age groups. The daily amount of water consumption is a function of climate, physical activity, culture, economics etc. The daily amount of water consumed by male adults, female adults, children and infants was 2.723, 2.129, 0.431 and 0.327 l/p-d, respectively (Agency, October, 2004).

## 3. Results

Concentration of ^222^Rn in Dassany, Bishe, Polour, Zam-Zam, Damavand, Crystal, Sivan and Koohrang Marks is 764±13, 794±15, 651±9, 879±17, 461±5, 754±13, 45±.56 and 771±14 Bq/m^3^, respectively. This divergence in concentration of ^222^Rn can be the result of differing water sources (surface or groundwater), production process, remaining time and water temperature (Ali et al., 2010; Ishikawa et al., 2005).

**Table 1:** Mean, range concentration of ^222^Rn (Bq/m^3^) in bottled waters in Bandar Abbas

Marks	Mean (Note 1)	range
Dassany	764±13	736-788
Bishe	794±15	621-710
Polour	651±9	739-806
Zam-Zam	879±17	856-901
Damavand	461±5	456-486
Crystal	754±13	728-779
Sivan	45±.56	0-56
Koohrang	771±14	702-864
Mean	641±9	0-901

**Figure 3:**
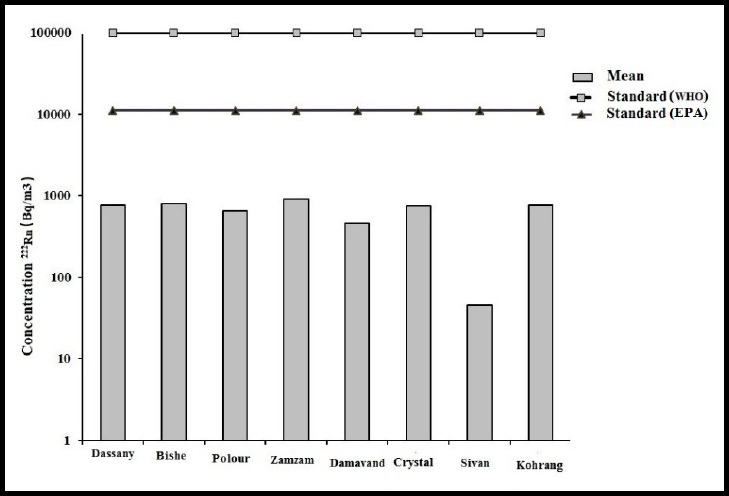
Comparison mean concentration of ^222^Rn across bottled water Marks with WHO and EPA standard limits

The mean and range concentration of ^222^Rn in all Marks were 641±9 and 0-901 Bq/m^3^, respectively. The range concentration of ^222^Rn in bottled waters used in Bandar Abbas is much lower than other cities of Iran and other countries. As mentioned previously, this concentration of ^222^Rn difference could be due to differing water source, temperature, and processing and storage time (Table 2) (Ishikawa et al., 2005). Concentration ^222^Rn of follows this order among the Marks: Zam-Zam>Bishe>Koohrang>Dassany>Crystal>Polour>Damavand>Sivan. Mean concentration of ^222^Rn in all the Marks was observed to be lower than the standards set by WHO (100×10^-3^ Bq/m^3^) and EPA (11×10^-3^ Bq/m^3^) (Figure 3).

**Table 2:** Comparison concentration of ^222^Rn in bottled waters of Bandar Abbas city with the results of national and world regions

	^222^Rn (Bq/l)	Country	References
Groundwater (wells)	0.89-35.44	Saudi Arabia	(Alabdula’aly, 1999)
Groundwater (wells)	0.95-36	Brazil	(Marques, dos Santos, & Geraldo, 2004)
Groundwater (wells)	0.7-31.7	Turkey	(Yalım, Sandıkcıoğlu, Ünal, & Orhun, 2007)
Tap water	0.91-12.58	Turkey	(Tarim et al., 2012)
Bottled water	0.91-1463	Serbia	(Todorovic et al., 2012)
Tap water	3.7	Iran (Tehran)	(Mowlavi, Shahbahrami, & Binesh, 2009)
Tap water	17.99	Iran (Neyshabour)	(Mowlavi et al., 2009)
Tap water	16.23	Iran (Mashhad)	(Mowlavi et al., 2009)
Tap water	3.4	Iran (Ramsar)	(Mowlavi et al., 2009)
Bottled water	0.641	Iran (Bandar Abbas)	This study

## 4. Discussion

**Figure 4:**
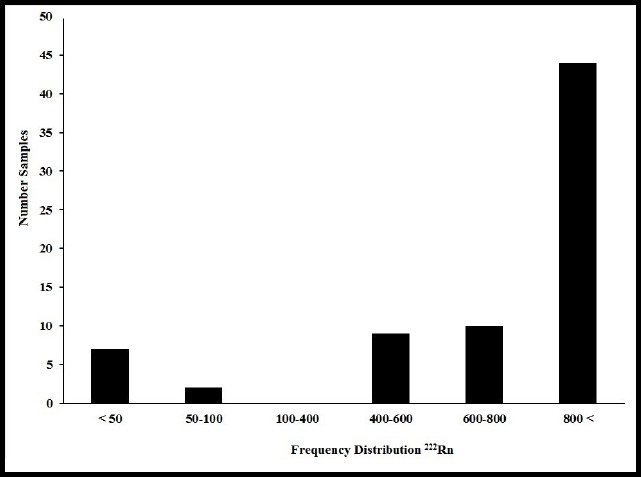
Frequency distribution concentration of ^222^Rn in bottled water samples of Bandar Abbas

The number of bottled water whose mean concentration of ^222^Rn is <50, 50-100, 100-400, 400-600, 600-800 and >800 Bq/m^3^ is 7, 2, 0, 10 and 44, respectively. The highest and lowest number of samples were observed in the concentration range of 100-400 Bq/m^3^ and above 800 Bq/m^3^, respectively (Figure 4).

**Figure 5:**
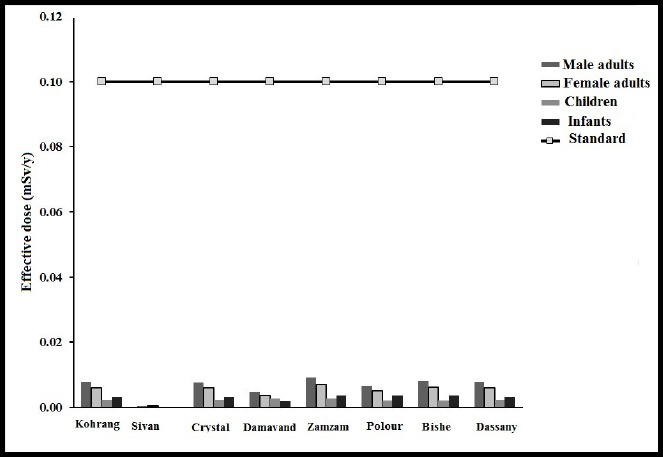
Comparison of the annually received effective dose in the 4 age groups (male and female adults, children and infants) with the standard limit

The effective dose received by the 4 age groups (male adults, female adults, children and infants) based on mean concentration of ^222^Rn in all the Marks (641±9 Bq/m^3^) was 0.0065, 0.0050, 0.0020 and 0.0027 mSv/y, respectively. The order of the effective dose received by the age groups is male adults>female adults>infants>children. The highest and lowest effective dose in male adults, female adults, children and infants belonged to Zam-Zam and Sivan Marks, respectively (Table 3). The proportion of the effective dose received by the 4 age groups to the standard dose (0.1 mSv/y) was 6.5%, 4.98%, 2.01% and 2.67%, respectively (Figure 5) (Risica & Grande, 2000; K. Somlai et al., 2007). The conversion coefficient was higher in children (26×10^-9^ Sv/Bq) and infants (35×10^-9^ Sv/Bq) than that of male and female adults (18×10^-9^ Sv/Bq). However, due to the more water consumption by adults (male adults: 2.7231/d; female adults: 2.1291/d), their annually received effective dose has been observed to be higher than children and infants. On the other hand, due to a bigger conversion coefficient, the effective dose received by infants is higher than children despite their less water consumption. The proportion of the effective dose received by male and female adults, children and infants to the standard limit (0.1 mSv/y) is in the following order among the famous Marks; Dassany: 7%, 5.9%, 2.4%, 3.1%, Bishe: 8.1%, 6.1%, 2.4%, 3.3%, Polour: 6.5%, 5%, 2%, 2.7%, Zam-Zam: 9.1%, 6.9%, 2.8%, 3.7%, Damavand: 4.6%, 3.5%, 1.4%, 1.9%, Crystal: 7.6%, 5.8%, 2.3%, 3.1%, Sivan: .45%, .34%, .14%, .18%, Koohrang: 7.8%, 5.9%, 2.4%, 3.2%, respectively. In order to determine whether there existed a significant divergence between the different age groups, one-way ANOVA was used. Since the p-value between the effective dose received in male and female adult groups was equal to 0.12, no significant difference can be said to exist between them (p value>0.05). Similarly, the p value=0.29 revealed no significant divergence between the effective dose received by children and infants. However, the p value=0.002 between the effective dose received by children and male adults, as well as the p value=0.015 between the effective dose received by children and female adults revealed a significant divergence between these groups. Similarly, the p value=0.002 between the effective dose received by infants and male adults as well as the p value=0.018 between infants and female adults revealed a significant divergence between these groups.

**Table 3:** Annually received effective dose of ^222^Rn (mSv/y) in the 4 age groups across the famous Marks in Bandar Abbas city

	Koohrang	Sivan	Crystal	Damavand	Zam-Zam	Polour	Bishe	Dassany	Mean
Male adults	0.0078	0.0005	0.0077	0.0047	0.0091	0.0066	0.0081	0.0078	0.0065
Female adults	0.0060	0.0003	0.0036	0.0036	0.0070	0.0051	0.0062	0.0059	0.005
Children	0.0024	0.0001	0.0015	0.0015	0.0028	0.0020	0.0020	0.0024	0.0020
Infants	0.0032	0.0002	0.0019	0.0019	0.0037	0.0033	0.0033	0.0032	0.0027

## 5. Conclusion

Mean concentration of ^222^Rn in all bottled water Marks was found to be less than the standard limits set by WHO and EPA. The highest and lowest concentration belonged to Zam-Zam and Sivan Marks. The highest and lowest proportion of effective dose to the standard effective dose was found to belong to male adults (Zam-Zam) and children (Sivan). The highest and lowest effective dose received was found to belong to male adults and children, respectively (p value <0.05). Also Infants were observed to receive a higher effective dose than children. The annually received effective dose through the ^222^Rn of bottled water in Bandar Abbas was lower than the standard limit (0.1 mSv/y) in all age groups.
